# Azetidinium Lead Halide Ruddlesden–Popper Phases

**DOI:** 10.3390/molecules26216474

**Published:** 2021-10-27

**Authors:** Jiyu Tian, Eli Zysman-Colman, Finlay D. Morrison

**Affiliations:** 1 EaStCHEM School of Chemistry, University of St Andrews, St Andrews KY16 9ST, UK; jt201@st-andrews.ac.uk; 2 Organic Semiconductor Centre, EaStCHEM School of Chemistry, University of St Andrews, St Andrews KY16 9ST, UK

**Keywords:** layered perovskite, bandgap tuning, azetidinium, Ruddlesden–Popper, structure-property relations

## Abstract

A family of Ruddlesden–Popper (*n* = 1) layered perovskite-related phases, Az_2_PbCl*_x_*Br_4−*x*_ with composition 0 ≤ *x* ≤ 4 were obtained using mechanosynthesis. These compounds are isostructural with K_2_NiF_4_ and therefore adopt the idealised *n* = 1 Ruddlesden–Popper structure. A linear variation in unit cell volume as a function of anion average radius is observed. A tunable bandgap is achieved, ranging from 2.81 to 3.43 eV, and the bandgap varies in a second-order polynomial relationship with the halide composition.

## 1. Introduction

Ruddlesden–Popper (R–P) phases are composed of layered perovskite structures with alternating layers of AMX_3_ perovskite and AX rock salt along the *c*-axis. They are described by the general formula A*_n_*_+1_M*_n_*X_3*n*+1_ (or A’_2_A”*_n_*_−1_M*_n_*X_3*n*+1_ in the case of two distinct A-cations), where *n* is a positive integer representing the number of perovskite layers that are separated by additional ‘A-cation excess’ rock-salt layers [1,2]. Importantly, the intergrowth rock salt layer means that the octahedra in the perovskite layers are aligned in the successive layers. In 1955, Balz and Plieth reported the first R–P phase layered structure K_2_NiF_4_ (*n* = 1) [3]. In 1957, Ruddlesden and Popper reported a series of layered structures in oxides, such as Sr_2_TiO_4_ and Ca_2_TiO_4_ [4]. Nowadays, the R–P phase is more commonly used to represent this type of layered perovskite structure and, increasingly, in organic–inorganic hybrid perovskites (OIHPs). Several families of layered OIHPs containing alternating layers of AMX_3_ perovskite and organic cations with structures similar to R–P phases have been reported. Such examples of layered OIHPs include BA_2_PbI_4_ (BA = C_4_H_9_NH_3_^+^) [5] and PEA_2_PbX_4_ (PEA = C_8_H_12_N^+^, X = Cl, Br, I), [6,7] in which the organic cations are too big to be accommodated in the cuboctahedral cavities of the 3D MX_6_ framework. Without the constraint of the size of the cuboctahedral cavities, a wider range of organic A-cations would be available for layered phases. In addition, by mixing large (A’) organic cations, such as those mentioned above, and small organic cations such as methylammonium (A” = MA), organic-inorganic hybrid materials with the general formula A’_2_A”*_n_*_−1_M*_n_*X_3*n*+1_ can be prepared [5,8]. They show good bandgap tunability by modifying the number of layers (*n*) of A”PbX_3_. Stoumpos et al. [5] reported orthorhombic crystal structures of BA_2_MA*_n_*_−1_Pb*_n_*X_3*n*+1_ (X = Br, I) with bandgaps changing progressively from 2.43 eV (*n* = 1) to 1.50 eV (*n* = ∞), with intermediate values of 2.17 eV (*n* = 2), 2.03 eV (*n* = 3) and 1.91 eV (*n* = 4). The thickness of the perovskite layer, *n*, in (BA)_2_(MA)*_n_*_−1_Pb*_n_*I_3*n*+1_ can be reasonably controlled by modifying the ratio of BA/MA cations in the precursor solutions. However, many so-called R–P phases reported in such compounds often do not have the required rock salt-structured interlayer between the 2D perovskite layers, resulting in an offset in the alignment of the perovskite blocks in successive layers. Such examples, therefore, do not conform to the definition of an R–P phase and are more correctly termed R–P-like OIHPs. Such R–P-like layered OIHPs have demonstrated higher stability when exposed to light, humidity and heat stress compared to 3D perovskite analogues, which are prone to unwanted phase transition under these test conditions [9,10]. For example, Ren et al. reported an R–P-like OIHPs solar cell material with general formula (MTEA)_2_(MA)_4_Pb_5_I_16_ (*n* = 5) which achieved a power conversion efficiency up to 17.8% [11]. Their cells retained over 85% of the initial efficiency after 1000 h operation time.

Azetidinium (Az^+^, (CH_2_)_3_NH_3_^+^) is a four-membered ring ammonium cation. In our previous study on mixed halide azetidinium lead perovskites, AzPbBr_3*−x*_X*_x_* (X = Cl or I), the structure progresses from 6H to 4H to 9R perovskite polytypes with varying halide composition from Cl^−^ to Br^−^ to I^−^ [12]. The fact that AzPbX_3_ (X = Cl or Br) forms a hexagonal perovskite rather than a cubic (3C) perovskite led to our study on mix-cation solid solutions of the form AzA”PbBr_3_, A” = MA^+^ or FA^+^ (FA^+^ = formamidinium). Such systems show only partial solid solutions and phase separation of the hexagonal and cubic forms; the extent of solid solution formation also depends on the synthesis route [13]. These studies also suggest that the cation radius of Az^+^ is ~310 pm, which is larger than the calculated cation radius of Az, *r*_Az_ = 250 pm (for comparison the reported radii for FA^+^ and MA^+^ are *r*_FA_ = 253 pm, *r*_MA_ = 217 pm [14], respectively). MA^+^ and FA^+^ are commonly used as A-site cations in OIHPs, and that adopt (pseudo-) cubic perovskite structures [15,16]. With our cation radius estimation that Az^+^ is larger than MA^+^ and FA^+^, Az_2_PbX_4_ (X = Cl, Br) are found to adopt a *n* = 1 R–P phase structure. The fact that Az^+^ can form a layered structure indicates that our estimation of its cation radius is more accurate than that from the computational calculation [13,14]. Furthermore, a family of mixed halide R–P phases, Az_2_PbCl*_x_*Br_4−*x*_ with composition 0 ≤ *x* ≤ 4 were prepared by mechanosynthesis and their structures and optical properties were analysed by powder X-ray diffraction (PXRD) and absorption spectroscopy, respectively. A linear variation in unit cell volume as a function of anion average radius is observed. The band gap was found to range from 2.81 to 3.43 eV, which varies as a second-order polynomial relationship with the halide composition.

## 2. Method

PbBr_2_ (98%) and PbCl_2_ (98%) were purchased from Alfa Aesar. Hydrobromic acid in water (48%) and AzCl (95%) were purchased from Fluorochem. All other reagents and solvents were obtained from commercial sources and used as received. AzBr were synthesised according to our previous study [17].

Preparation of Az_2_PbCl*_x_*Br_4−*x*_ solid solutions with 0 ≤ *x* ≤ 4 (in *x* = 0.67 increments) was carried out by mechanosynthesis. Appropriate molar ratios of dry AzX and PbX_2_ (AzX:PbX_2_ = 2:1, X = Cl or Br) were ground together in a Fritsch Pulverisette planetary ball mill at 600 rpm for 1 h using 60 cm^3^ Teflon pots and high-wear-resistant zirconia media (nine 10 mm diameter spheres). Az_2_PbBr_4_ samples could also be obtained by hand grinding AzBr and PbBr_2_ in an agate mortar and pestle for 25 min.

PXRD was carried out using a PANalytical Empyrean diffractometer with Cu K_α1_ (λ = 1.5406 Å). Rietveld refinements of PXRD data using GSAS [18] were used to confirm phase formation and for the determination of lattice parameters.

Optical properties were determined from solid-state absorption spectra recorded using a Shimadzu UV-2600 spectrophotometer and bandgaps were calculated by plotting (*αhν*)^2^(cm^−1^·eV)^2^ with *hν*(eV) according to the Tauc method, in which *α*, *h* and *ν* stand for absorbance, Planck’s constant and incident light frequency.

## 3. Results

**Figure 1:**
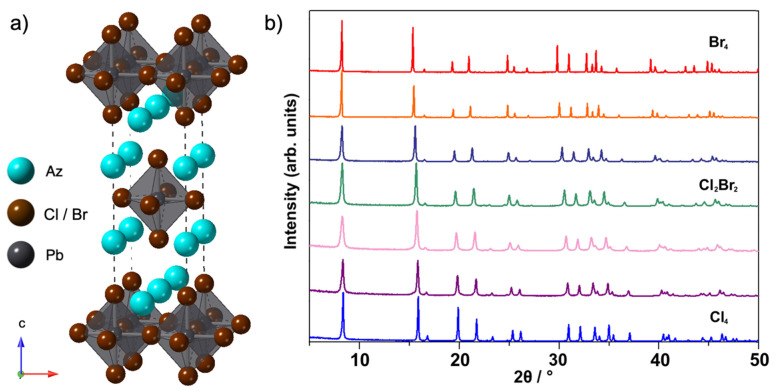
(**a**) *n* = 1 Ruddlesden–Popper (R–P) phase of Az_2_PbX_4_ (X = Cl, Br) showing alternating AzPbX_3_ perovskite and AzX rock salt layers along the *c*-axis, (**b**) PXRD data of mix-halide layered R–P phases: Az_2_PbCl*_x_*Br_4−*x*_ with composition 0 ≤ *x* ≤ 4 prepared by mechanosynthesis.

The PXRD data for Az_2_PbCl*_x_*Br_4−*x*_ with compositions ranging from 0 ≤ *x* ≤ 4 were prepared by mechanosynthesis and are shown in Figure 1b. The structures of these samples were determined to be R–P *n* = 1 phase in the *I*4/*mmm* space group (Figure 1a). The theoretical diffraction pattern of the tetragonal R–P phase is shown in Figure S1. Characteristic peaks of the R–P phase show systematic peak shifts to higher 2*θ* angle from Az_2_PbBr_4_ to Az_2_PbCl_4_, which indicate the lattice parameters decreased with more Cl content in the solid solution. The Az^+^ cations, which are represented as solid spheres situated at the centre of electron density, form rock salt layers with the X^−^ anions. Synthesis from solution is preferred when manufacturing devices because solutions can be easily processed into thin films by spin-coating and blade-coating methods compared to bulk powder [19]. Thus, precipitation synthesis of Az_2_PbX_4_ (X = Cl, Br) were also attempted (synthetic details included in the supporting information) and their PXRD data are shown in Figure S2. Although the precipitated samples contain additional phase(s) associated with additional peaks (e.g., at 6° and 11°) and have yet to be assigned to a structure. Ganguli [20] reported an empirical prediction that possible R–P phase structures are associated with a ratio of A-site and metal cation radii (*r*_A_/*r*_M_) in the range of 1.7 to 2.4. As discussed in our previous study [12], our estimation of the cation radius of Az^+^ (~310 pm) differs from that calculated (250 pm) [14]. The *r*_Az_/*r*_Pb_ calculated using our estimated radius is 2.60, while that using the literature value [14] is 2.10.

Unfortunately, our attempts to synthesise single-phase Az_2_PbI_4_ were unsuccessful. The PXRD of mechanosynthesised Az_2_PbI_4_ is shown in Figure S3. In addition to the R–P phase, there are evident amounts of 9R AzPbI_3_ phase [12,21] and the relative intensity of this phase increased with increased ball mill grinding time (1 to 3 h). PXRD of the Az_2_PbI_4_ sample obtained from a hand grinding synthesis showed that this method can increase the proportion of R–P phase in the samples, evidenced by the increased relative intensity of peaks associated with the R–P phase, but the presence of the 9R phase persisted across all samples. These results indicate that the 9R phase is the more stable phase compared to the R–P phase for the iodide analogue It is likely that the activation energy for the transformation of azetidinium lead iodide from a layered phase to the 9R phase is low.

**Figure 2:**
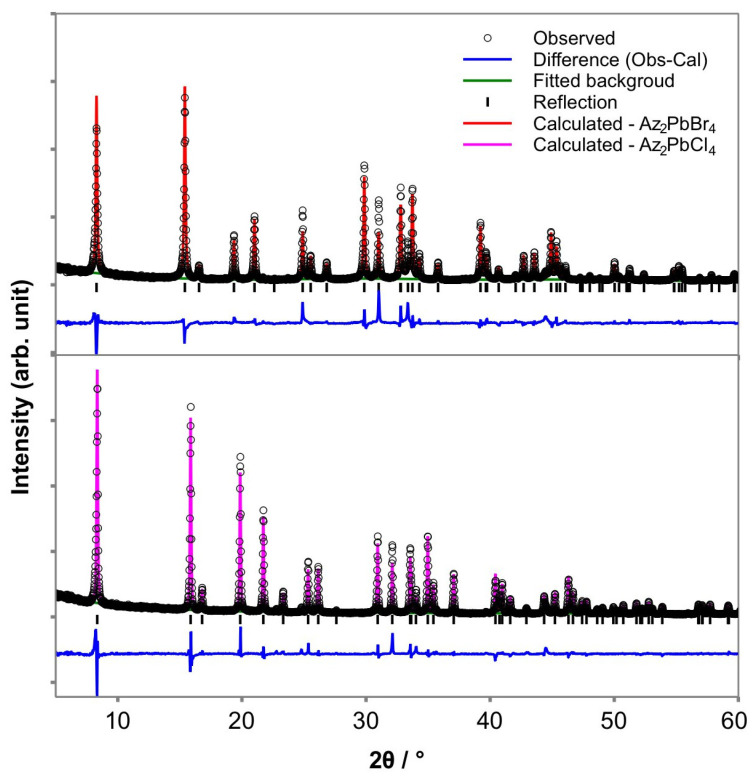
Rietveld refinement of PXRD data in *I*4/*mmm* space group of Az_2_PbX_4_, X = Br (top) and Cl (bottom) obtained from mechanosynthesis with observed data (open circles), calculated data (red line for Br and magenta line for Cl), background (green lines), reflection positions (black bars) and difference plots (blue lines).

For simplicity, Rietveld refinements were carried out by replacing the organic Az^+^ cations with Mn^2+^, as they have similar electron densities. Figure 2 shows an example of the PXRD data refinement of Az_2_PbX_4_ (X = Cl, Br) samples obtained from the ball mill mechanosynthesis. The refined lattice parameters of Az_2_PbBr_4_ are *a* = 5.993(6) Å and *c* = 21.501(1) Å, with goodness-of-fit parameters *χ*^2^ = 10.21 and *wR_p_* = 0.115, while those of Az_2_PbCl_4_ are *a* = 5.765(0) Å and *c* = 21.027(2) Å, with goodness-of-fit parameters *χ*^2^ = 7.20 and *wR_p_* = 0.102. The difference between the organic moieties and Mn^2+^, which is associated with their actual atomic position and thermal motion, is one possible reason for such high *χ*^2^ values for both refinements and may be responsible for the differences in the peak shape and intensities shown. Single crystal diffraction analysis is required for detailed structural analysis, including accurate atoms positions (particularly of the Az^+^ cation), however, this would require preparation of sufficiently large single crystals which are challenging by this mechanosynthesis route. Nevertheless, it is clear from the rudimentary Rietveld analysis of the PXRD data that all peaks are accounted for and that the PXRD unambiguously show the formation of *n* = 1 R–P materials. In addition, as the peaks positions can be determined accurately the unit cell dimensions are reliable.

**Figure 3:**
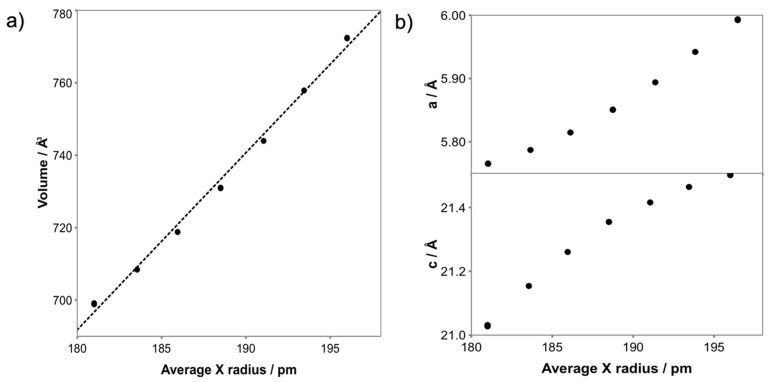
(**a**) Cell volume, (**b**) lattice parameters as a function of average halide anion radius for *n* = 1 R–P phases Az_2_PbCl*_x_*Br_4−*x*_ (0 ≤ *x* ≤ 4) as determined from Rietveld refinement of PXRD data.

To study the mixed-halide solid solutions Az_2_PbCl*_x_*Br_4__−*x*_, the lattice parameters of each mechanosynthesised composition were determined by Rietveld refinement of PXRD data. The cell volume of these R–P phases varies linearly as a function of the average anion radius, Figure 3a (the average anion radius was calculated using *r*_Br_ = 196 pm and *r*_Cl_ = 181 pm according to Shannon [22]). This linear variation is expected in accordance with Vegard’s law. The lattice parameters *a* and *c*, on the other hand, show a nonlinear relationship with the average anion radius (Figure 3b), which suggests anisotropic expansion/contraction along the *a*- and *c*-axis. The larger expansion in *a* is consistent with the increased X anion radius which affords a larger void for the Az^+^ cation, resulting in less required expansion in the interlayer spacing. Based on the analysis using Mn^2+^ as a proxy for Az^+^ we have no information regarding any orientation or dynamics of the Az^+^ cation.

One of the benefits of mechanosynthesis is that all materials are retained during the reaction, so the overall starting composition must be retained in the post-reaction compound(s). By inference, any product(s) must have the nominal starting composition. While we do not have direct compositional analysis, the PXRD results, Figure 2, clearly show that the product formed is entirely *n* = 1 R–P phase. It has been reported that the actual composition shows a good match with the nominal composition in the mechanosynthesis of OIHPs [23,24]. Thus, the halide compositions of Az_2_PbCl*_x_*Br_4−*x*_ are calculated according to the molar ratios of the raw materials (nominal composition).

**Figure 4:**
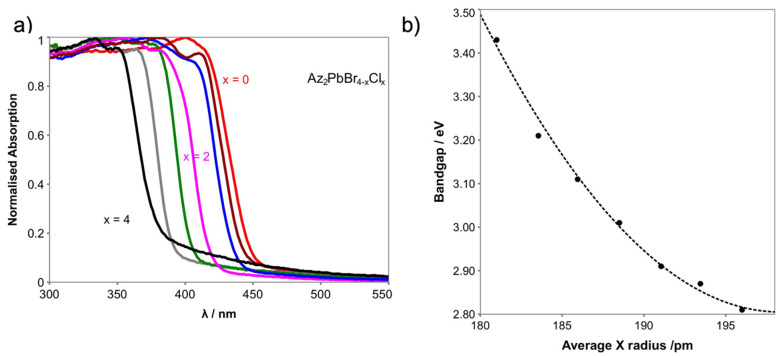
(**a**) Absorption spectra; (**b**) bandgap determination from the absorption spectra of samples Az_2_PbCl*_x_*Br_4−*x*_ with composition 0 ≤ *x* ≤ 4 plotted as a function of average halide anion radius.

The optical properties of Az_2_PbCl*_x_*Br_4__−*x*_ (0 ≤ *x* ≤ 4) solid solutions were studied by absorption spectroscopy (Figure 4a). The absorption onsets are systematically red-shifted from ca. 386 nm (Az_2_PbCl_4_) to ca. 457 nm (Az_2_PbBr_4_) with increasing average anion size (from Cl^−^ to Br^−^). The bandgaps of Az_2_PbCl_4_ and Az_2_PbBr_4_ are calculated to be 3.43 and 2.81 eV, which are the same (within error) as the bandgap of the 6H hexagonal perovskite AzPbCl_3_ (3.43 eV) and AzPbBr_3_ (2.81 eV) [12]. However, unlike the linear variation in the 6H AzPbX_3_ (X^−^ = Cl^−^, Br^−^), the bandgap of layered R–P Az_2_PbX_4_ (X = Cl, Br) shows a bowing with the average anion radius (Figure 4b). The bowing effect [25,26] simply describes the deviation of the measured band gap in continuous solid solutions from the values expected by linear interpolation of the end member values. Band gap bowing is often fitted to a second-order polynomial to account for the divergence from linearity, with a bowing parameter *b* as the binominal coefficient of the fitting Equation (1): [26]

(1) $$E_{g}\left(x\right)=\left(1-x\right)E_{g|\left(x=0\right)}+xE_{g|\left(x=1\right)}-bx\left(1-x\right)$$

The bowing parameter, *b*, of the mechanosynthesised mixed halide layered Az_2_PbCl*_x_*Br_4−*x*_ (0 ≤ *x* ≤ 4) is 0.47 with a goodness-of-fit R^2^ value of 0.995. The bowing parameter of mixed halide OIHPs are usually smaller, variously reported as 7 × 10^−4^ to 0.33 for MAPbBr_3*−x*_X*_x_* (X = Cl or I), [27,28] compared to the bowing parameters (0.4 to 1.33) found for other mixed metal perovskite systems such as MA_3_(Sb_1−*x*_Bi*_x_*)I_9_ (0.4 for Bi rich region and 1.3 for Sb rich region) and 1.06 for MA(Pb_1−*x*_Sn*_x_*)I_3_ [25,26,29].

## 4. Conclusions

*n* = 1 Ruddlesden–Popper (R–P) layered perovskite phases were successfully obtained by mechanosynthesis in the mixed halide solid solution Az_2_PbCl*_x_*Br_4−*x*_ with composition 0 ≤ *x* ≤ 4. Az_2_PbX_4_(X = Cl, Br) was determined to be the conventional R–P *n* = 1 (K_2_NiF_4_) structure with a space group of *I*4/*mmm*. A linear variation in unit cell volume as a function of anion average radius is observed. The band gap of the R–P phases Az_2_PbCl_4_ and Az_2_PbBr_4_ are determined to be 3.43 and 2.81 eV, which is the same (within error) as the bandgap of 6H hexagonal perovskite AzPbCl_3_ (3.43 eV) and AzPbBr_3_ (2.81 eV) [12]. A bowing effect with a bowing parameter of 0.47 is observed in the band gap-composition relationship of R–P layered mixed halide solid solutions, compared to the linear relationship observed in the 6H hexagonal perovskite.

## Data Availability

The research data supporting this publication can be accessed at https://doi.org/10.17630/fd5aab9b-fced-4926-afee-5eb56e2e6a5e (accessed on 15 October 2021).

## Supplementary Materials

The following are available online. Supporting Information data include synthetic details of precipitation synthesis of Az_2_PbX_4_(X = Cl, Br) (Figures S1 and S2) and synthesis of Az_2_PbI_4_ (Figure S3). Also, include selected crystallographic data obtained powder X-ray diffraction of samples prepared by mechanosynthesis (Table S1).

## Sample Availability

Samples of the Az_2_PbCl*_x_*Br_4−*x*_ with composition 0 ≤ *x* ≤ 4 are available from the authors.
