# Supplementary material for: Azetidinium Lead Halide Ruddlesden–Popper Phases
Source: Molecules. 2021 Oct 27;26(21):6474. doi: 10.3390/molecules26216474 (PMC8588525; doi:10.3390/molecules26216474)
Supplement: Supplementary file 1 [file molecules-26-06474-s001.zip › molecules-1421572-supplementary.pdf]

Supporting Information

Azetidinium lead halide Ruddlesden-Popper phases

Jiyu Tian[1,2], Eli Zysman-Colman[2,*] and Finlay D. Morrison[1,*]

[1]EaStCHEM School of Chemistry, University of St Andrews, St Andrews, Fife, KY16 9ST, United Kingdom.

[2]Organic Semiconductor Centre, EaStCHEM School of Chemistry, University of St Andrews, St Andrews, Fife, KY16 9ST, United Kingdom.

[1,2]jt201@st-andrews.ac.uk, Corresponding authors: [2,*]eli.zysman-colman@st-andrews.ac.uk; [1,*]finlay.morrison@st-andrews.ac.uk

Table of Contents

Theoretical PXRD patterns of Az_2PbX_4 (X = Cl, Br) 1

Precipitation synthesis of Az_2PbX_4 (X = Cl, Br) 2

Attempted synthesis of Az_2PbI_4 3

References 5

Theoretical PXRD patterns of Az_2PbX_4 (X = Cl, Br)

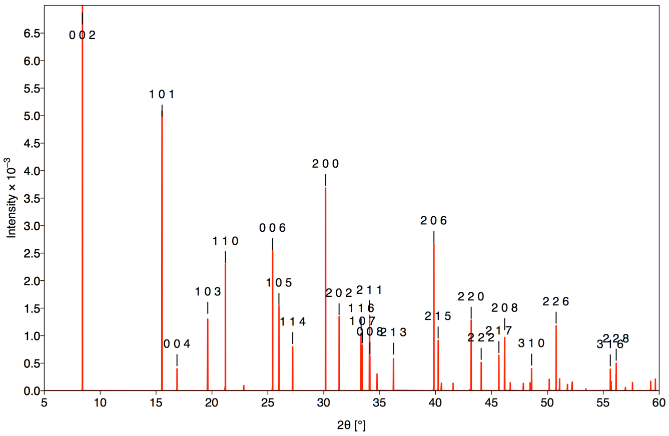

Figure S1. Theoretical PXRD patterns of Az_2PbBr_4 with indexing of major peaks.

Precipitation synthesis of Az_2PbX_4 (X = Cl, Br)

Az_2PbCl_4 samples were prepared by dissolving AzCl and $PbCl_2$ (2:1) in DMSO (2 mL, 0.4 M $PbCl_2$) at room temperature and in air. The reaction mixture was stirred for 1 h whereupon clear solutions were obtained. Acetone (15 mL) was added slowly into the solution and the vial was shaken for 1 min and then left to stand for 10 min before vacuum filtration. The resulting powders were washed with 2×10 mL acetone and then dried under vacuum for 24 h. The obtained samples were white powders.

Precipitation synthesis attempts of Az_2PbBr_4 samples were carried out by mixing appropriate molar ratios AzBr and $PbBr_2$ (2:1) in DMF/DMSO (3:1, 0.3 M $PbBr_2$) at room temperature. Sonication and stirring were applied to aid in the dissolution of the reagents. DCM (20 mL) was added slowly into the solution and the vial was shaken for 1 min and then left to stand for 2 min before vacuum filtration. The resulting powders were washed with 2×10 mL DCM and then dried under vacuum for 24 h.

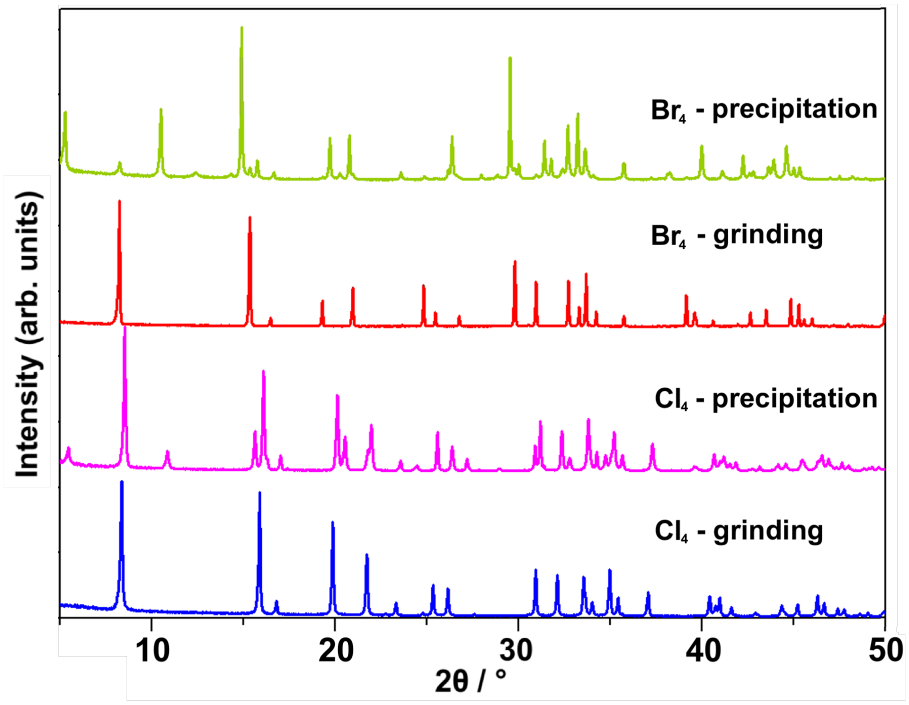

Figure S2. PXRD data of Az_2PbX_4 (X = Cl, Br) prepared by mechanosynthesis and precipitation synthesis.

Attempted synthesis of Az_2PbI_4

AzI was synthesised according to our previous study.[1] Appropriate molar ratios of dry AzI and PbI_2 ($AzI:PbI_2$ = 2:1) were ground together in a Fritsch Pulverisette planetary ball mill at 600 rpm for 1 h either single or multiple times (e.g., Az_2PbI_4-ballmill_3h indicates 3 × 1-hour grinding). 60 cm^3 Teflon pots and high-wear-resistant zirconia media, which are nine zirconia grinding media 10 mm diameter spheres, were used in the synthesis. Az_2PbI_4 samples were attempted by hand grinding. AzI and PbI_2 mixtures were ground by hand with a mortar and pestle for 4 ×15 min.

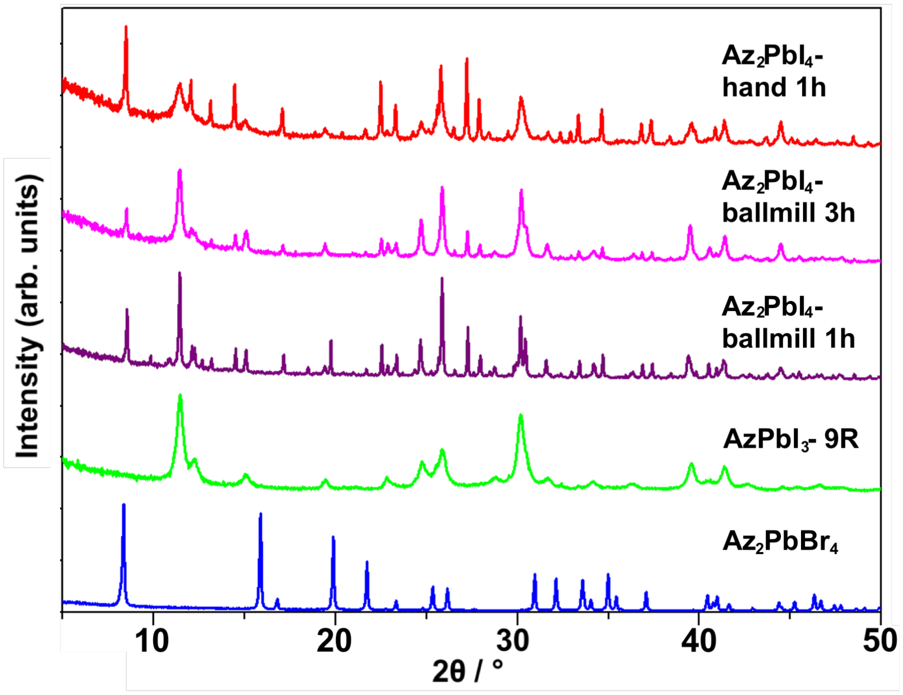

Figure S3. PXRD data of attempted syntheses of Az_2PbI_4 prepared by hand grinding and ball mill mechanosynthesis. For reference, PXRD data for previously prepared R-P Az_2PbBr_4 and 9R perovskite $AzPbI_3$ phases[2] prepared by mechanosynthesis are also shown.

Table **S1**. Selected crystallographic data obtained powder X-ray diffraction of samples prepared by mechanosynthesis.

	Az_2PbCl_4	$Az_2PbBr_{0.67}Cl_{3.33}$	$Az_2PbBr_{1.33}Cl_{2.67}$	$Az_2PbBr_2Cl_2$	$Az_2PbBr_{2.67}Cl_{1.33}$	$Az_2PbBr_{0.67}Cl_{3.33}$	Az_2PbBr_4
temperature [K]	293	293	293	293	293	293	293
empirical formula	$C_6H_{16}Cl_4N_2Pb$	$C_6H_{16}Br_{0.67}Cl_{3.33}N_2Pb$	$C_6H_{16}Br_{1.33}Cl_{2.67}N_2Pb$	$C_6H_{16}Br_2Cl_2N_2Pb$	$C_6H_{16}Br_{2.67}Cl_{1.33}N_2Pb$	$C_6H_{16}Br_{0.67}Cl_{3.33}N_2Pb$	$C_6H_{16}Br_4N_2Pb$
fw	554.12	568.94	583.75	598.57	613.39	628.20	643.02
crystal description	Colorless prism	Colorless prism	Colorless prism	Yellow prism	Yellow prism	Yellow prism	Yellow prism
space group	*I*4/*mmc*	*I*4/*mmc*	*I*4/*mmc*	*I*4/*mmc*	*I*4/*mmc*	*I*4/*mmc*	*I*4/*mmc*
a [Å]	5.765(0)	5.786(9)	5.814(5)	5.850(5)	5.893(9)	5.942(1)	5.993(6)
c [Å]	21.027(2)	21.154(0)	21.260(6)	21.354(6)	21.415(8)	21.464(3)	21.501(1)
vol $[Å]^3$	698.85(2)	708.41(8)	718.80(1)	730.93(3)	743.95(1)	757.86(5)	772.41(1)
Z	2	2	2	2	2	2	2
GOF	0.1052	0.0868	0.0779	0.0824	0.0934	0.1664	0.1370
χ_2	7.203	5.984	5.380	5.471	8.026	14.49	10.23
wR_2 (all data)	0.1019	0.0955	0.0853	0.0807	0.0906	0.1409	0.1155

References

1. Tian, J.; Zysman-Colman, E.; Morrison, F.D. Compositional Variation in Hybrid Organic-Inorganic Lead Halide Perovskites: Kinetically versus Thermodynamically Controlled Synthesis. *Chem. Mater.* **2021**, *33*, 3650–3659, doi:10.1021/acs.chemmater.1c00470.

2. Tian, J.; Cordes, D.B.; Slawin, A.M.Z.; Zysman-Colman, E.; Morrison, F.D. Progressive Polytypism and Bandgap Tuning in Azetidinium Lead Halide Perovskites. *Inorg. Chem.* **2021**, *60*, 12247–12254, doi:10.1021/acs.inorgchem.1c01425.
